# Endurance and avoidance response patterns in pain patients: Application of action control theory in pain research

**DOI:** 10.1371/journal.pone.0248875

**Published:** 2021-03-25

**Authors:** Jana Buchmann, Nicola Baumann, Karin Meng, Jana Semrau, Julius Kuhl, Klaus Pfeifer, Miguel Kazén, Heiner Vogel, Hermann Faller

**Affiliations:** 1 Department I—Psychology, University of Trier, Trier, Germany; 2 Institute of Clinical Epidemiology and Biometry, University of Würzburg, Würzburg, Germany; 3 Department of Sport Science and Sport, Friedrich-Alexander-University Erlangen-Nürnberg, Erlangen, Germany; 4 Department of Psychology, University of Osnabrück, Osnabrück, Germany; 5 Section of Medical Psychology and Psychotherapy, University of Würzburg, Würzburg, Germany; Unviersity of Sheffield, UNITED KINGDOM

## Abstract

**Background:**

Identifying pain-related response patterns and understanding functional mechanisms of symptom formation and recovery are important for improving treatment.

**Objectives:**

We aimed to replicate pain-related avoidance-endurance response patterns associated with the Fear-Avoidance Model, and its extension, the Avoidance-Endurance Model, and examined their differences in secondary measures of stress, action control (i.e., dispositional action vs. state orientation), coping, and health.

**Methods:**

Latent profile analysis (LPA) was conducted on self-report data from 536 patients with chronic non-specific low back pain at the beginning of an inpatient rehabilitation program. Measures of stress (i.e., pain, life stress) and action control were analyzed as covariates regarding their influence on the formation of different pain response profiles. Measures of coping and health were examined as dependent variables.

**Results:**

Partially in line with our assumptions, we found three pain response profiles of distress-avoidance, eustress-endurance, and low-endurance responses that are depending on the level of perceived stress and action control. Distress-avoidance responders emerged as the most burdened, dysfunctional patient group concerning measures of stress, action control, maladaptive coping, and health. Eustress-endurance responders showed one of the highest levels of action versus state orientation, as well as the highest levels of adaptive coping and physical activity. Low-endurance responders reported lower levels of stress as well as equal levels of action versus state orientation, maladaptive coping, and health compared to eustress-endurance responders; however, equally low levels of adaptive coping and physical activity compared to distress-avoidance responders.

**Conclusions:**

Apart from the partially supported assumptions of the Fear-Avoidance and Avoidance-Endurance Model, perceived stress and dispositional action versus state orientation may play a crucial role in the formation of pain-related avoidance-endurance response patterns that vary in degree of adaptiveness. Results suggest tailoring interventions based on behavioral and functional analysis of pain responses in order to more effectively improve patients quality of life.

## Introduction

Psychosocial risk and resilience factors play an important role in multidisciplinary biopsychosocial rehabilitation (MBR) of chronic non-specific low back pain, which is low back pain lasting more than 12 weeks without a specific pathoanatomical diagnosis [1–4]. Therefore, identifying pain-related response patterns and understanding underlying functional mechanisms of symptom formation and recovery are essential steps for tailoring interventions to increase the effectiveness of treatment [4,5].

Analyzing subgroups of patients by means of cluster analysis has a long tradition in pain research (e.g. [6–13]). In comparison to traditional clustering techniques (e.g., hierarchical and k-means cluster analyses), however, latent class or profile analyses constitute model-based clustering techniques that allow determining probabilities of group membership, the significant number of latent groups, goodness of model fit, and including covariates that are supposed to influence group membership. Up to date, only few studies based on traditional cluster analysis exist that specifically examined pain-related avoidance- and endurance responses in patients with (sub-)acute and chronic low back pain [14–19]. The focus of this study thus was to examine the appropriate number of pain-related avoidance and endurance response patterns by means of latent profile analysis as well as their primary and secondary characteristics. In addition, we analyzed the influence of stress and action control as covariates in accordance with Action Control Theory [20–22].

In the past two decades of cognitive-behavioral pain research, there has been growing interest in avoidance- and endurance-related pain response patterns and theoretical explanations of development and maintenance, or recovery from (chronic) pain (e.g., [15,16,19,23–27]). In the following, we explicate two prominent cognitive-behavioral models of pain chronification and associated studies concerning proposed cognitive, emotional and behavioral pain responses, and their health-related consequences. In addition, we specify how Action Control Theory may contribute to a better understanding of these processes and related empirical findings.

### Fear-avoidance model

As one of the most prominent models, the *Fear-Avoidance Model* (FAM) suggests two diametrically opposite paths with patterns of cognitive, emotional and behavioral pain responses, which facilitate either the development of chronic pain, disability and depression (FAM-1), or else recovery (FAM-2):

FAM-1: Fear-avoidance responses, and
FAM-2: No-fear confrontational responses

Maladaptive fear-avoidance responses consist of catastrophizing, anxiety/depression, and avoidance of physical and social activities and are associated with elevated pain, disability, and depression, which is attributed to a kinesiophobia-like pathology [25]. Thereby, individuals interpret aversive pain experiences as a threatening sign of harm, injury, or a serious physical illness (catastrophizing), which elicits fear of pain and pain-related movements. Elevated pain-related fears, in turn, lead to rapid increases in pain perception (hypervigilance) and avoidance of pain-related movements. In the short-term, associated decreases in pain and emotional distress may reinforce avoidance behavior. In the long-term, however, increased avoidance and reduction of physical and social activities may facilitate physical deconditioning, accumulate loss of social reinforcement, and finally lead to a disuse syndrome [25,28]. In contrast, no-fear confrontational pain responses without catastrophizing and conditioned avoidance behavior are assumed to prevent chronic pain and facilitate recovery [25].

Empirical evidence, however, partially contradicts the assumptions of the existence of only one fear-avoidance response pattern and only one single path to chronic pain as proposed by the FAM. For example, in line with the FAM, there is evidence supporting the assumption that catastrophizing and/or pain-related fear/anxiety are associated with avoidance of pain-related movements, changes in musculoskeletal functioning and flexion, and increases in pain, distress, and disability [25,27,29–33]. In addition, Smeets and colleagues found evidence for reduced aerobic fitness in patients with chronic low back pain compared to a healthy control group [34]. Other RCT studies, however, found little or no evidence for disuse and physical deconditioning in patients with chronic low back pain [28,35,36]. Likewise, several findings contradict the assumption that catastrophizing or pain-related fear/anxiety are associated with reduced physical activity, physical deconditioning, or poor outcomes [25,34,37,38].

### Avoidance-endurance model

One possible explanation for these inconsistent results is that both anxious and non-anxious individuals often respond with persistence despite pain instead of fear-avoidance behavior. Consistent with this explanation, endurance-related pain responses were shown to occur as often or even more frequently as fear-avoidance responses [26,39]. Accordingly, the *Avoidance-Endurance Model* (AEM) proposes excessive endurance responses despite pain to be responsible for increases and maintenance of pain and disability. Thus, the AEM includes fear-avoidance responses (AEM-1), as does the FAM, but further suggests maladaptive distress-/eustress-endurance responses despite pain that consist of thought suppression, persistence behavior despite pain, and either anxiety/depression (AEM-2) or else positive mood (AEM-3) [40]. In contrast to these first three response patterns, the AEM proposes adaptive responses (AEM-4) to be characterized by low endurance and avoidance responses, and to prevent the development of chronic pain due to an adequate balance between activity and relaxation:

AEM-1: Fear-avoidance responses,
AEM-2: Distress-endurance responses,
AEM-3: Eustress-endurance responses,
AEM-4: Adaptive responses.

As a consequence of pain and pain-related fear/anxiety, people may not only try to avoid pain-related movements or interrupt activity in general (“flight” or “freezing” responses). Especially under chronic exposure to stress (e.g., external control, work-related demands, and ongoing valued activities), they may even try harder to control the intrusive outbreaks of pain and inner conflicts, or avoid anticipated loss and negative outcomes by means of suppression and persistence responses despite pain (“fight” response). This may be especially reflected in distress-endurance responses [39,41].

Hasenbring and colleagues assumed that eustress-endurance responders, in comparison to distress-endurance responders, show less disability because they are more active and better withstand pain-related interruptions of daily activities [42]. In the long-term, however, the AEM generally proposes endurance responses to be detrimental due to excessive overactivity, prolonged postural strain/overuse, and suboptimal motor control [26,39]. These are supposed to cause overload and new (micro-)injuries of soft tissues, which lead to increases and maintenance of pain and disability. Supporting these assumptions, studies based on three or four AEM patterns partially found endurance responders to show higher levels of constant strain positions, accelerometer-based physical activity, pain, and/or disability than did adaptive responders [42–44], as well as higher accelerometer-based physical activity and pain than did fear-avoidance responders [44]. Cluster-analytical studies (see Table 1), however, found no differences between AEM-like response clusters in other measures of physical activity [15,16].

**Table 1:** Overview of cluster-analytical studies based on pain-related avoidance-endurance responses.

Study samples	Cluster variables/analysis	Cluster solutions (discussion)
Grebner et al. [14]: *N* = 82 patients with recurrent acute radicular pain (pre-surgical)	11 avoidance-endurance subscales (KPI/**AEQ**), depression **(ADS)**; **HCA Ward**	**4 clusters (AEM-like)**: FAR, DER, EER, AR (R confounded with C variables)
Fehrmann et al. [15]: *N* = 137 patients with chronic low back pain (pre-treatment)	2 endurance subscales (**AEQ**), 1 subscale of SF-36; **HCA Ward**	**4 clusters (AEM-like):** FAR, DER, EER, AR (without avoidance responses, 15 of 23 indices for 3-cluster solution, R confounded with C variables)
McCracken & Samuel [16]: *N* = 276 patients with chronic pain ^1^	Avoidance, confronting (task/pain persistence), pacing (PARQ); **HCA Ward**	**4 clusters (AEM-like):** Avoiders, extreme, medium cyclers (~pacing), doers
Esteve et al. [17]: *N* = 276 patients with chronic musculoskeletal pain ^1^	Pain/activity avoidance, task-contingent, pain-contingent, excessive persistence, pacing (APS); **HCA Ward**	**4 clusters (AEM-like):** avoiders (with pacing), extreme, medium cyclers (~pacing), doers
Holldorf et al. [18]: *N* = 268 patients with subacute radicular pain (post-surgical, pre-treatment)	Pain-related self-instructions (FSS), fear-avoidance beliefs (FABQ-D), coping (FKV-LIS-SE), anxiety, depression (HADS-D), health status (SF-36); **HCA Ward**	**2 clusters (FAM-like):** Depressive avoiders, eustress endurance (R confounded with C variables)
Cane et al. [19]: *N* = 842/307 pain patients (pre-/post-treatment)	Subscales for avoidant, overdoing, pacing (POAM-P); **HCA Ward**	**Pre: 2 clusters (FAM-like)**: Avoidance-pacing, persistence **Post: 4 clusters (AEM-like):** Avoidance-pacing, persistence, mixed, pacing
Rabey et al. [45]; *N* = 294 patients with chronic low back pain ^1^	2 endurance subscales (**AEQ**), catastrophizing (PCS), acceptance (CPAQ), self-efficacy (PSEQ), fear-avoidance-beliefs (FABQ), stress, anxiety, depression, (DASS)**; LCA**	**3 clusters:** With differing median levels of stress, EA responses, coping, and mental health (R confounded with S and C variables, no pairwise comparisons)

Note: HCA = hierarchical cluster analysis, LCA = latent class analysis; FAR = fear-avoidance responses, DER = distress-endurance responses, EER = eustress-endurance responses, AR = adaptive responses; S = stimulus variables, O = organismic variables, R = response variables, C = consequences, according to the S-O-R-C scheme for behavioral and functional analyses [46] (i.e., adapted for functional analyses by discriminating content-focused response and function-focused organismic variables according to Kuhl [47])

^1^ no information concerning pre-/post-treatment status.

Most of the respective cluster-analytical studies further reported less pain, better health and better functioning in pain patients with non-avoidant confronting/eustress-endurance and adaptive responses than in patients with distress-endurance and/or avoidance responses [15–17,19]. In addition, eustress-endurance responders, similar to low-endurance responders, showed a better therapy prognosis than did fear-avoidance or distress-endurance responders [15,24]. Findings suggest that patients with eustress-endurance and adaptive responses may have better self-regulatory abilities contributing to better coping, health, and therapy prognosis than do patients with fear-avoidance or distress-endurance responses. In particular, stress research suggests eustress responses to be a result of increased volitional capabilities to cope with life stress, as well as subjective evaluations of stress (responses) as controllable or gain-related, associated with positive feelings, self-efficacy, and better health [48–52].

### Action control theory

Against this background, we seek to better understand the underlying stress-dependent mechanisms of *progression* in terms of facilitated top-down control of action according to someone’s needs and specific to global goals versus *regression* to lower levels of functioning in terms of stimulus-driven automatic, impulsive or conditioned behavior under external control [47,50]. To this end, we employ the *Action Control Theory* (ACT), which proposes *action versus state orientation* as an organismic resilience versus risk factor (O) moderating or mediating symptom formation, maintenance, and recovery from life stress [20,22,47,53–56]. State orientation is defined as a disposition for excessive *preoccupation* with uncontrollable, failure-related thoughts (rumination) instead of action-oriented disengagement; and for *hesitation* instead of action-oriented initiative concerning difficult intentions to reach an alternatively intended goal [57,58]. Thus, dispositional action (vs. state) orientation captures the ability to maintain or even enhance executive volitional functioning under threatening and demanding conditions, including pain [20,22,50,59–62].

In the face of elevated life stress (i.e., threats and demands), action- as compared to state-oriented individuals can better intuitively self-regulate emotions, that is down-regulate negative emotions through self-relaxation and up-regulate positive emotions through self-motivation [53,56,63–65]. A better intuitive emotion regulation is associated with a better top-down control of goal-oriented processing, such as self-discrimination and coherence judgements, updating working memory to disengage from unwanted or unattainable goals, inhibiting counter-intentional automatized processes, as well as initiating and persisting in (pro-)active coping activities [20,22,53,61–63,66–71].

Therefore, and due to more context-sensitive forms of self-confrontative coping, action (vs. state-) oriented individuals are predisposed to a better adjustment to pain- and work-related life stress [20,22,50,72]. Consistent with this assumption, state- compared to action-oriented patients reported more pain and distress, negative thoughts, monitoring of their wounds, and less successful distraction efforts after a surgery of hernia [22]. Moreover, Luka-Krausgrill, Wurmthaler, Wiesheu and Becker found that depressed patients with chronic pain showed higher levels of life stress and state orientation than did non-depressed patients.

Little is known, however, about the stress-dependent formation of specific pain response patterns in patients with chronic low back pain. In contrast to the assumptions of the AEM, for example, patients with low-endurance responses may be confronted with less pain- and work-related life stress (i.e. threats and demands) that usually may trigger avoidance and endurance responses. Moreover, in comparison to Action Control Theory, FAM and AEM do not make any assumptions about different self-regulatory abilities that may predispose patients to develop more or less adequate strategies to cope with different levels of pain- and work-related life stress. The present study aimed to additionally address these two aspects by means of covariate analyses.

### Objective 1: Replicating FAM-/AEM-like pain response profiles

To our knowledge, this is the first study that aimed to identify the (appropriate number of) latent profiles underlying avoidance and endurance responses in patients with chronic nonspecific low back pain. We thereby examined the characteristics of FAM-/AEM-like pain response profiles by using cognitive, emotional, and behavioral avoidance-endurance response measures (i.e., pain response measures as primary indicator variables for latent profile analysis).

According to FAM and AEM, we expected two distinct profiles of diametrically opposing fear-avoidance and no-fear (confrontational) eustress-endurance responses. In line with AEM, we further expected two less distinct and opposite profiles of distress-endurance and low-endurance responses (analogous to the low-risk adaptive AEM pattern). Together, we aimed to replicate the following four FAM- plus AEM-like pain response profiles in patients with chronic non-specific low back pain:

1. *Fear-avoidance responses (FAR)*, with high levels of avoidance responses and low levels of endurance responses,
2. *Distress-endurance responses (DER)*, with high levels of both distress-endurance and avoidance responses,
3. *Eustress-endurance responses (EER)*, with high levels of eustress-/endurance responses and low levels of avoidance responses,
4. *Low*-*endurance responses (LER)*, with low levels of both endurance and avoidance responses.

### Objective 2: Differences between pain response profiles in secondary measures

Our second objective was to validate pain response profiles with regard to secondary S-O-R-C measures, which is a prerequisite for an integrative behavioral and functional analysis that combines Action Control Theory with cognitive-behavioral S-(O)-R-C models of pain chronification (i.e., the FAM and AEM). The S-O-R-C scheme by Kanfer and colleagues differentiates psychological parameters concerning certain stimuli (S), mediating or moderating organismic variables (O), cognitive, emotional and behavioral responses (R), and their consequences (C). We therefore examined whether distinct pain response profiles (R) differ in secondary measures of stress (S), action control (O), coping (R), and health (C). In comparison to secondary measures of stress (i.e., pain and life stress) and action control, which are supposed to be independent variables (i.e., covariates) that influence the formation of pain response profiles, secondary measures of mal-/adaptive coping and health are supposed to be dependent variables (i.e., additional pain response characteristics and health outcomes that are assumed to be associated with or determined by the underlying pain response profiles).

#### Covariates

In line with Action Control Theory, we mainly expected significant differences between two pairs of opposite response profiles based on different levels of stress and action control: Pain patients with high levels of pain and life stress (i.e., perceived threats and demands as potential triggers of avoidance and endurance responses) as well as low action control due to low self-regulatory abilities to cope with stress were assumed to either show marked fear-avoidance, or distress-endurance responses to pain (i.e., regressive “flight” or “fight” responses). In contrast, pain patients with better self-regulatory abilities to cope with stress, who perceive higher levels of pain and life stress, rather may develop a more successful eustress-endurance response profile. Finally, patients who experience lower levels of pain and life stress presumably associated with higher self-regulatory ability to cope with stress may show a profile of low-endurance responses (analogous to the low-risk adaptive AEM pattern).

Together, we assumed that patients with fear-avoidance and those with distress-endurances responses suffer from higher levels of pain and life stress as well as lower levels of action versus state orientation, and would show the poorest adaptive coping, and health as compared to patients with eustress-endurance or low-endurance responses, respectively. In contrast, we expected that eustress-endurance and low-endurance responders are predisposed by higher levels of action versus state orientation, whereas low-endurance responders, however, would show lower levels of pain and life stress as compared to the other three response profiles. Moreover, eustress-endurance responders are assumed to show the highest levels of physical activity as well as equal levels of mal-/adaptive coping and health as compared to patients with low-endurance responses.

## Method

### Participants

The current study is a secondary analysis of data from *n* = 536 patients with chronic non-specific low back pain from three German rehabilitation centers. Participants attended an inpatient orthopedic rehabilitation program and were recruited for one year. Exclusion criteria of the primary study were age below 18 or above 65 years, inadequate German language ability, severe impairment of vision or hearing, a poor health state preventing patients from participation in additional patient education and filling out questionnaires, severe co-morbid psychiatric disorders, and an ongoing retirement application [1]. All participants provided written informed consent. The primary study was approved by the Ethics Committee of the University of Erlangen-Nürnberg and performed following the Declaration of Helsinki.

### Measures

This study is based on self-report data of patients at the beginning of an inpatient rehabilitation program. Besides the variables described below, the questionnaire included demographic and social-medical information (see Table 2).

**Table 2:** Frequencies, means and standard deviations in socio-demographical, medical, primary response, and secondary variables.

Variables	*M (SD*)
Age (years)	49.1 (8.1)
Sex, female *n* (%)	275 (51.3)
Higher-level school education (> 10 years) *n* (%)	112 (20.9)
Currently in paid employment *n* (%)	481 (89.7)
Medical visits last 6 months	5.3 (5.6)
Days off work due to back pain last 6 months	19.9 (38.5)
Catastrophizing	0.9 (1.1)
Help-/hopelessness	2.2 (1.2)
Anxiety/depression	2.2 (1.2)
Avoidance of physical activity	3.1 (1.0)
Avoidance of social activity	1.9 (1.1)
Thought suppression	3.5 (1.5)
Positive mood	3.4 (1.2)
Humor/distraction	3.2 (1.0)
Task/pain persistence	3.5 (0.9)
Pain	5.8 (1.6)
Life stress	7.3 (5.3)
Failure-related action orientation	5.7 (3.4)
Prospective action orientation	7.4 (3.2)
Subjective competence	16.2 (4.5)
Cognitive restructuring	14.3 (4.7)
Rumination	2.1 (0.9)
Pain-related fear (somatic focus)	5.0 (3.4)
Physical Activity	8.1 (5.7)
Depression	6.5 (4.3)
Mental health	47.1 (11.3)	
Physical health	37.7 (8.6)

Note: *M* = unstandardized mean, *SD* = standard deviation.

#### Primary response measures

Cognitive, emotional, and behavioral avoidance-endurance responses within the last two weeks was assessed by nine subscales of the Avoidance-Endurance Questionnaire (AEQ) [40]. Avoidance-related subscales were *catastrophizing* (3 items), *help-/hopelessness* (9 items), *anxiety/depression* (7 items), *avoidance of physical activity* (12 items), and *social activity* (10 items). Endurance-related subscales were *anxiety/depression* (7 items) for distress-endurance, *thought suppression* (4 items) and *task/pain persistence* (14 items) for both eustress- and distress-endurance, as well as *positive mood* (3 items) and *humor/distraction* (10 items) for eustress-endurance responses. Half of the items related to behavioral AEQ subscales address responses under mild pain, the other half responses under severe pain. Analyses of internal consistency yielded Cronbach’s *α*s = .84 to .92. Higher mean values indicate higher frequencies of avoidance and endurance responses to pain.

#### Secondary measures

To assess *pain*, patients were asked to rate their current, worst and average pain intensity during the last week on an eleven-point numerical rating scale (NRS; 3 items, Cronbach’s α = .77) adapted from Nagel and colleagues [73]. A mean score for pain was computed, with higher mean values indicating higher pain. *Life stress* was measured by an adopted scale from the short version of the Volitional Components Inventory (VCI) [74]. Due to high correlations between the original two subscales ‘threats’ and ‘demands’ (*r* = .79, *p* < .001), we created one scale *‘threats and demands’* (8 items, Cronbach’s α = .91), with higher sum scores indicating higher levels of life stress.

To assess dispositional *action versus state orientation*, the Action Control Scale (ACS-90) [58] was administered. The ACS-90 consists of two subscales (12 items each) with a dual response format capturing *failure-related action versus state orientation* (AOF; disengagement vs. preoccupation, Cronbach´s α = .82); and *prospective action versus state orientation* (AOP; initiative vs. hesitation, Cronbach´s α = .79). Higher sum scores indicate higher levels of action orientation.

Adaptive coping was assessed by the subscales *subjective competence* and *cognitive restructuring* (4 items, Cronbach´s α = .80/.73) from the German Pain Management Questionnaire (FESV) [75]. Maladaptive coping in terms of *rumination* and *pain-related fear* was assessed by the subscales rumination (4 items, Cronbach´s α = .83) from the Pain Catastrophizing Scale (PCS) [76], and fear of pain/(re-)injury–somatic focus (5 items, Cronbach’s α = .79) from the German version of Tampa Scale of Kinesiophobia (TSK-GV) [77]. Higher mean values indicate higher levels of adaptive and maladaptive coping, respectively. Moreover, *physical activity* was assessed by eight items from the Freiburg Questionnaire of Physical Activity (FFkA) [78]. It measures basic (e.g. stairs climbed, walking, cycling), leisure (e.g. gardening) and sports activities in the last week or month. Item scores were summarized to build an overall sum score expressed in hours per week.

*Depression* was assessed by the eight-item version of the depression module of the Patient Health Questionnaire (PHQ-8) [79]. It measures the severity of depressive symptoms over the past two weeks, with higher sum scores indicating more severe depression (Cronbach’s α = .83). *Mental* and *physical health* were measured by the German version of the SF-12 Health Survey [80], with higher scores indicating a better health state.

### Statistical analysis

Statistical analyses were carried out using SPSS 23 (IBM Corp., Armonk, NY), and Mplus^TM^, version 7 (Muthén & Muthén, Los Angeles, CA) [81]. Missing data were imputed using a multiple imputation procedure. Means and standard deviations for quantitative variables as well as frequencies and percentages for categorical variables were computed to describe patient characteristics and relations between primary indicator and secondary variables (see also S1–S3 Tables). Moreover, SPSS was used to depict and evaluate most of the characteristics of and differences between the final subgroups.

Latent profile analysis (LPA) [82–85] was conducted by Mplus to classify patients into homogeneous subgroups (i.e., latent profiles), who additionally share a meaningful and interpretable pattern of pain-related cognitive, emotional, and behavioral avoidance-endurance responses. In comparison to latent class analysis (LCA), which is used to identify subgroups based on categorical indicator variables, LPA is used to identify subgroups of patients based on continuous indicator variables [84] such as individual mean values on the AEQ subscales.

The LPA was done in two steps: First, the best set of starting values was identified and replicated to evaluating fit indices for each of five models ranging from one to five latent profiles without covariates (unconditional model) [84,85]. Thereby, one thousand random starts were calculated to prevent local solutions. Second, four covariates that were assumed to influence the formation of different response profiles (i.e., pain, life stress, failure-related and prospective action versus state orientation) were evaluated in the LPA for the selected best fitting model [84]. Each covariate was evaluated separately with respect to their contribution to the fit of the final model, as well as differences between pairs of latent profiles by using logistic regression analysis (i.e., with covariates as independent variables, and latent profile variable as dependent variable).

Estimation of latent profile membership and covariance analysis were carried out with robust maximum-likelihood (MLR) using the expectation-maximum (EM) algorithm [86]. Concerning model adequacy, the minimum average probability of latent class/profile membership should be >.80 [85]. The most appropriate number of latent profiles was identified by evaluating several fit indices, such as the log likelihood value (LL), Akaike’s Information Criterion (AIC), Bayesian Information Criterion (BIC), Sample-Adjusted BIC (SABIC), and entropy as well as by using the adjusted Lo-Mendell Ruben test (LMR) and bootstrap likelihood ratio test (BLRT). Whereas LL, AIC, BIC, and SABIC indicate increasing model fit by decreasing values, entropy is as measure of classification uncertainty with well-fitting models indicated by values ≥.80 [84]. In addition, a well-fitting model has to make sense conceptually, and the estimated profiles should differ as hypothesized in secondary variables that were not used as primary indicator variables to generate the model [87].

After identifying the latent profile solution that best fits the data, differences between pain response profiles in primary and secondary response characteristics and more distal health outcomes were evaluated using univariate analyses of variance (ANOVAs) and pairwise multiple mean comparisons (i.e., with the latent profile variable as independent variable, and secondary response and health outcomes as dependent variables). For all statistical tests, *p*-values of *p* < .05 were considered statistically significant.

## Results

### Descriptives

In the total sample (*n* = 536), participants’ mean age was 49 years, with ages ranging from 19 to 64 years. Fifty-one percent (*n* = 275) of the sample were women, and 90% were in paid employment at the beginning of rehabilitation (for more information, see Table 2).

### Objective 1: Replicating FAM-/AEM-like pain response profiles

Latent profile analysis and LMR tests revealed that the three-profile model had a significantly better model fit than the two-profile model (see Table 3). Against our hypotheses, however, LMR test revealed that the fit of the four-profile model did not significantly improve as compared to the three-profile model. Moreover, aside from similar profiles like those of the three-profile solution, the four-profile solution only revealed a fourth less distinct and opposite eustress-endurance-related pain response profile. Thus, we selected the more parsimonious three-profile model since it revealed better LMR test results and interpretability than the other models with four or five response profiles, respectively. A good model adequacy of the three-profile solution is further indicated by the average probabilities for most likely latent profile membership, which amounted to .93 (profile 1) and .90 (profiles 2, 3).

**Table 3:** LPA model fit summary.

Model Profile/s	Log likelihood	AIC	BIC	SABIC	Entropy	Smallest class %	LMR p-value	LMR meaning	BLRT p-value	BLRT meaning
1	-7427.25	14890.49	14967.61	14910.47			-	-	-	-
2	-7053.37	14162.73	14282.69	14193.81	0.78	42	***	2 > 1	***	2 > 1
**3**	**-6869.16**	**13814.31**	**13977.11**	**13856.49**	**0.80**	**26**	***	**3 > 2**	***	**3 > 2**
4	-6770.32	13636.64	13842.28	13689.91	0.80	17	n.s.	4 < 3	***	4 > 3
5	-6696.42	13508.83	13757.31	13573.20	0.80	10	n.s.	5 < 4	***	5 > 4

Note. *n* = 536; LPA = latent profile analysis; AIC = Akaike’s Information Criterion; BIC = Bayesian Information Criterion; SABIC = Sample-Adjusted BIC; LMR = Lo-Mendell Ruben; BLRT = bootstrap likelihood ratio test; The LMR test and the BLRT compare the current model to a model with k– 1 profiles, significant test results support the retention of the more complex solution

*** = *p* < .001.

**Fig 1:**
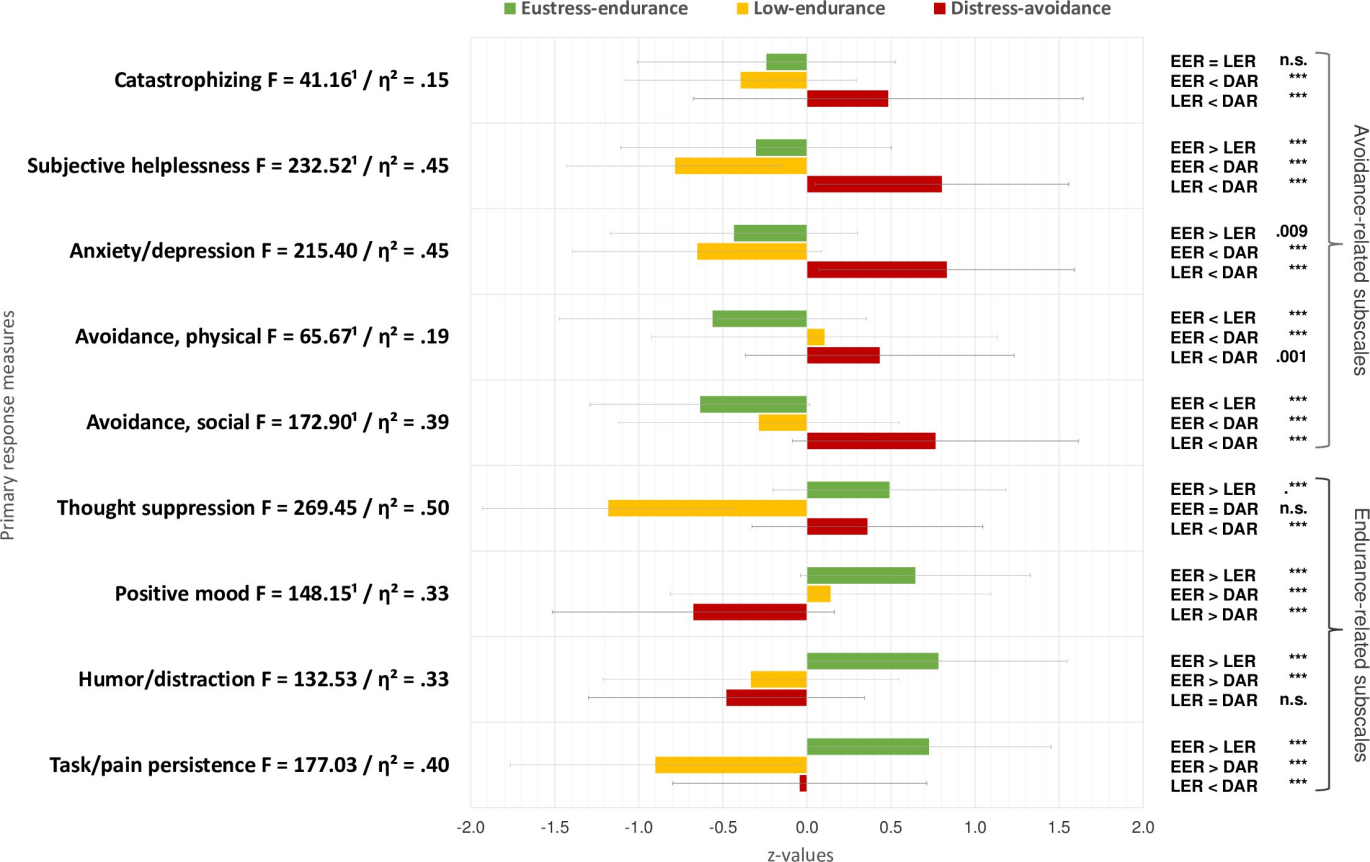
Z-standardized means, standard deviations, overall (ANOVAs) and multiple mean comparisons of pain response profiles on primary response measures. Note: *n*_EER_ = 187, 35%, *n*_LER_ = 141, 26%, *n*_DAR_ = 208, 39%; EER = Eustress-endurance, LER = Low-endurance, DAR = Distress-avoidance responders; ^1^ = Welsh test; all overall *p* < .001; *** = *p* < .001; *η^2^* classification of effect sizes by Cohen (1988): *η^2^* = .01, small, *η^2^* = .06, medium, *η^2^* = .14, large.

Partially in line with our hypotheses, LPA revealed the following three response profiles (for more information on means, standard deviations, significance of differences and relative position, see Tables 4 and S3, and Fig 1):

1. *Distress-avoidance responses* (DAR: n = 208, 39%) with higher levels of catastrophizing, help-/hopelessness, anxiety/depression, avoidance behavior, and thought suppression, medium levels of task/pain persistence, as well as lower levels of positive mood and humor/distraction;
2. *Eustress-endurance responses* (EER: n = 187, 35%) with lower to medium levels of catastrophizing, help-/hopelessness, anxiety/depression and avoidance behavior; as well as higher levels of thought suppression and task/pain persistence, positive mood and humor/distraction;
3. *Low-endurance responses* (LER: n = 141, 26%) with lower levels of catastrophizing, help-/hopelessness, and anxiety/depression, medium levels of avoidance behavior, as well as lower levels of thought suppression and task/pain persistence, positive mood and humor/distraction.

**Table 4:** Means and standard deviations of pain response profiles in primary response measures.

Primary response measures	EER	LER	DAR
	M (SD)	M (SD)	M (SD)
Catastrophizing	0.63 (0.87)	0.45 (0.78)	1.45 (1.31)
Help-/hopelessness	1.81 (0.95)	1.24 (0.76)	3.12 (0.89)
Anxiety/depression	1.70 (0.89)	1.43 (0.90)	3.24 (0.93)
Avoidance physical activity	2.57 (0.91)	3.23 (1.02)	3.55 (0.79)
Avoidance social activity	1.22 (0.74)	1.62 (0.94)	2.80 (0.96)
Thought suppression	4.18 (1.01)	1.74 (1.09)	3.98 (1.00)
Positive mood	4.24 (0.85)	3.61 (1.19)	2.59 (1.04)
Humor/distraction	3.93 (0.74)	2.85 (0.85)	2.71 (0.79)
Task/pain persistence	4.21 (0.68)	2.70 (0.81)	3.50 (0.70)

Note: M = unstandardized mean, SD = standard deviation; EER = Eustress-endurance, LER = Low-endurance, DAR = Distress-avoidance responders.

#### Overall effects

Univariate ANOVAs yielded significant differences (p < .001) with large effect-sizes (partial η^2^) indicating substantial contribution***s*** of each measure to discriminating between the profiles (see Fig 1).

#### Multiple mean comparisons

Mostly as expected, we found two almost diametrically opposing response profiles of distress-avoidance and eustress-endurance responses. Patients with distress-avoidance responses showed higher scores on all avoidance-related subscales and lower scores on the endurance-related subscales compared to patients with eustress-endurance responses; except for equal levels of thought suppression (see also Fig 1). Almost in line with our assumptions, we further found a third profile of low-avoidance-endurance responses with the lowest levels of catastrophizing, help-/hopelessness, anxiety/depression, thought suppression, and task/pain persistence compared to both other pain response profiles.

Unexpectedly, we did not find a distinct cluster of pure fear-avoidance responses in neither the three- nor the four-profile-solutions.

### Objective 2: Differences between pain response profiles in secondary measures

#### Covariates

**Fig 2:**
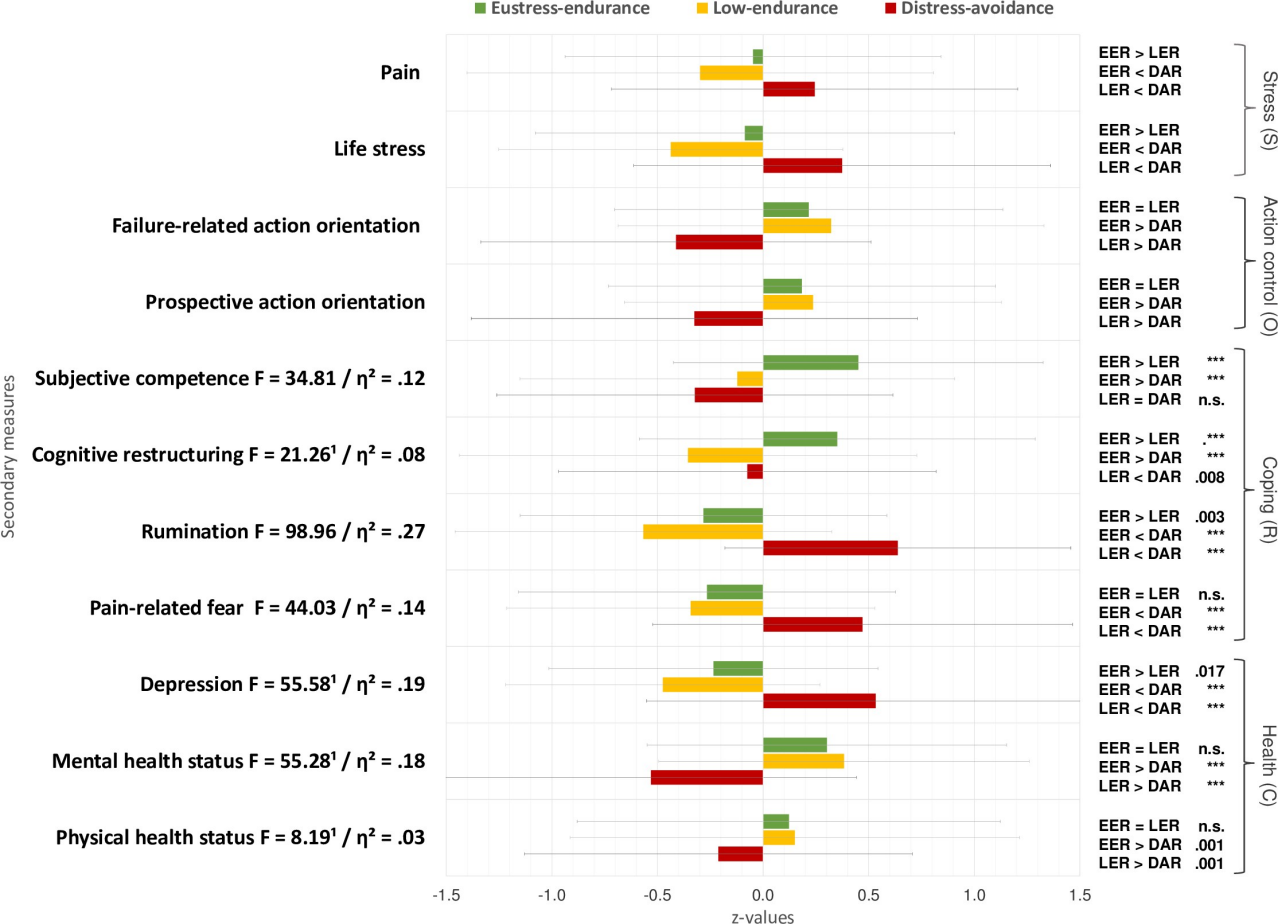
Z-standardized means, standard deviations, overall (ANOVAs) and pairwise mean comparisons of pain response profiles on secondary measures. Note: EER = Eustress-endurance, LER = Low-endurance, DAR = Distress-avoidance responders; ^1^ = Welsh test; all overall *p* < .001; *** = *p* < .001; η^2^ classification of effect sizes by Cohen [88]: *η^2^* = .01, small, *η^2^* = .06, medium, *η^2^* = .14, large effect.

In line with our hypotheses, covariate analyses mostly yielded significant differences between the latent profiles for each covariate, except for non-significant differences between eustress-endurance and low-endurance responders on measures of action (vs. state) orientation (see Table 5). Almost as expected, distress-avoidance responders showed the highest levels of pain and life stress as well as the lowest levels of action (vs. state) orientation as compared to both other response profiles. Moreover, low-endurance responders revealed the lowest levels of pain and life stress as well as similar levels of action (vs. state) orientation as compared to eustress-endurance responders (see Fig 2).

**Table 5:** LPA model fit summary for the three-profile solution (model 3) with four covariates including p-values from logistic regression analysis.

Covariate	Log likelihood	AIC	BIC	SABIC	Entropy	Smallest class %	*P*	*P*	*P*
							EER-LER	EER-DAR	LER-DAR
Pain	-6852.32	13784.63	13956.00	13829.02	0.80	25	.025	.002	***
LS	-6833.19	13746.38	13917.74	13790.77	0.80	27	.004	***	***
AOF	-6830.27	13740.54	13911.90	13784.93	0.80	26	n.s.	***	***
AOP	-6850.39	13780.79	13952.15	13825.18	0.81	27	n.s.	***	***

Note. n = 536; LPA = latent profile analysis; AIC = Akaike’s Information Criterion; BIC = Bayesian Information Criterion; SABIC = Sample-Adjusted BIC; LS = life stress, AOF = failure-related action orientation, AOP = prospective action orientation; *p* = *p*-value

*** = p < .001.

#### Overall effects on dependent secondary measures

As further expected, univariate ANOVAs yielded significant differences between the profiles on all secondary measures of coping including physical activity (*F* = 10.41, *p* = .000, *η^2^* = .04) and health (see also Fig 2).

#### Multiple mean comparisons

In line with our assumptions, patients with eustress-endurance and those with low-endurance responses showed lower levels of life stress, maladaptive coping, depression, and a better health state, as well as higher levels of failure-related action orientation and physical activity compared to patients with distress-avoidance responses. Moreover, eustress-endurance responders reported the highest levels of adaptive coping and physical activity compared to both other profiles (*p*_EER > DAR_ = .000, *p*_EER > LER_ = .023), whereas low-endurance responders did not differ in their levels of physical activity compared to distress-avoidance responders [*M* (*SD*): EER = 9.46 (5.59), LER = 8.02 (6.11), DAR = 6.87 (5.36)]. Against our assumptions, patients with low- endurance responses showed (one of) the lowest levels of adaptive coping (i.e., subjective competence and cognitive restructuring), similar to patients with distress-avoidance responses.

## Discussion

To our knowledge, this is the first study that aimed to replicate FAM-/AEM-like pain response patterns in patients with chronic non-specific low back pain by means of latent profile analysis, and validated pain response profiles by using measures of stress, action control, coping and health. Results were partly consistent with prior cluster-analytical studies, as well as the Fear-Avoidance Model (FAM) and Avoidance-Endurance Model (AEM). However, in line with Action Control Theory (ACT), findings also extend prior research in terms of a new endurance-avoidance typology (EAT).

### Characteristics of FAM-/AEM-like pain response profiles

Partially in line with FAM and/or AEM, we found two distinct opposite pain response profiles of distress-avoidance and eustress-endurance responses, and one partially opposite profile of low-avoidance-endurance responses:

1. *Distress-avoidance responses (DAR)* with higher levels of avoidance, medium to higher levels of distress-endurance, and mostly lower levels of eustress-endurance responses,
2. *Eustress-endurance responses (EER)* with higher levels of eustress-/endurance responses, and lower to medium levels of avoidance responses,
3. *Low- endurance responses (LER)* with lower to medium levels of avoidance, and lower levels of endurance responses.

Similar to clusters of depressive avoidance responders, mixed, or extreme cyclers, which were found in prior cluster-analytical studies [16–19], only one compound profile of mixed distress-avoidance responders (DAR) consistently emerged that showed both higher levels of fear-avoidance and medium levels of distress-endurance responses as compared to both other response profiles. Against our assumptions according to the FAM and AEM, we did not find a distinct profile of pure fear-avoidance responses. This is a new finding, which was not reported in previous traditional cluster-analytical studies based on subscales of the Avoidance-Endurance Questionnaire [14,15].

Noteworthy, we found that life stress-related measures of threats and demands as potential triggers of fear-avoidance and distress-endurance responses were highly intercorrelated in the current sample of patients with chronic low back pain (see S2 Table). Presumably, recurring pain as an archetypical aversive stressor is both threatening (because it normally signals harm) and demanding (because it interrupts ongoing activities and demands attention) (see also [89,90]). In the course of pain chronification, it may increasingly threaten an individual’s health needs and work/life goals. Likewise, the challenges and demands to fulfill health needs and attain personal work/life goals also increase with elevated pain and pain-related disability. In addition, chronic pain may induce inner conflicts between individual health needs (e.g., reducing pain by reducing activity) and work/life goals (e.g., persisting and reaching personal goals in spite of pain).

Beyond the FAM and AEM, this supports the assumption that the paths explaining the development and maintenance of chronic pain through (partial or graded) *disuse*/deconditioning and *misuse*/overload and associated avoidance-endurance responses are multiply interconnected and reinforcing each other. Basically, this may be the case because individuals differ in their self-regulatory ability (i.e., their action vs. state orientation) to flexibly cope with pain- and work-related life stress, unrealistic intentions as well as conflicts in a balanced and sustainable manner [20,22,41,50]. Importantly, this also provides more individual explanations for some of the inconsistent findings questioning the FAM. For example, there is little evidence for general disuse and physical deconditioning among patients with chronic low back pain compared to healthy control groups [28,35,36]. However, when pain patients show periods of reduced activity and avoidance of specific movements as compared to their former habitual activity levels, this may at least reduce their physical fitness and (muscle-specific) load-bearing capacity in relation to their former individual work load and regular occupational demands (in contrast to excessive general disuse and deconditioning as proposed by the FAM). As a consequence, when patients try to face again their individual daily life challenges and occupational work load as usual (in contrast to excessive misuse as proposed by the AEM), they probably overload their muscles and other soft tissues, leading to new (micro-)injuries, increased pain and disability.

Partially in line with mixed distress-endurance responses (DER) as proposed by the AEM, and according to Action Control Theory, our finding of distress-avoidance responses (DAR) thus suggest that at least short reactance periods of increased efforts to endure in habitual work load and persist in ongoing activities despite pain may intermingle with avoidance of pain-related movements and reduced activity in terms of fight-or-flight/freezing responses [20,21,39]. These, however, may primarily be based on avoidance motivation (e.g., avoiding conflicts, pain/-related threats, or a loss of control over work-related demands) accompanied by a stress-dependent loss of volitional top-down control [21,41]. This loss of top-down control is characterized by a regression to more rigid forms of low-level processing such as coping based on habitual, impulsive, or conditioned stimulus-response connections as it was suggested for state- (vs. action-) oriented individuals in the face of increased life stress [20,47,50,62]. This is in line with other research suggesting that thought suppression associated with endurance responses despite pain may reflect attempts to control or avoid unwanted thoughts and is often followed by rebound effects (i.e., counter-intentional intrusions of unwanted thoughts), especially under demanding conditions [91,92].

In contrast, positive feedback and self-affirmation were shown to eliminate rebound effects [93]. This may be especially the case in eustress-endurance responders (EER), who consistently showed equal levels of thought suppression compared to distress-avoidance responders as well as the highest levels of positive mood, humor/distraction, task persistence, and subjective competence compared to both other response types. According to Action Control Theory, findings suggest that pro-/active, more successful suppression and distraction efforts in action-oriented eustress-endurance responders may be a consequence of better emotion regulation, and attentional disengagement, positive emotions facilitating the enactment of difficult intentions and cognitive or automatic goal shielding against counter-intentional impulses and action alternatives [21,22,39,61,70,94]. Volitional facilitation of difficult intentions in action- as compared to state-oriented individuals, for example, was shown as a consequence of positive primes in Stroop experiments, which were especially related to achievement motivation [70,95]. This suggests that more action-oriented eustress-endurance responders may better maintain top-down control under increased pain-related demands resulting in more successful suppression and distraction efforts. Further research, however, is needed to confirm these hypotheses.

### Differences between pain response profiles in secondary measures

Mostly as expected, patients with distress-avoidance responses (DAR) emerged as the patient group with the most severe levels of stress, maladaptive coping, and bad health. This is largely in line with meta-analytical data concerning the impact of activity avoidance on pain and (psychological) functioning as well as other cluster-analytical studies that also identified avoidance responders, mixed, or extreme cyclers as (one of) the most dysfunctional patient groups [15–19,23].

Moreover, in line with Action Control Theory, distress-avoidance responders emerged as the most state-oriented patient group. One the one hand, this was indicated for failure-related action (vs. state) orientation, which captures the ability to down-regulate negative emotions through self-relaxation and disengage from unrealistic intentions and unwanted negative thoughts [57,58,63,67]. The inability to down-regulate negative emotions further is associated with reduced access to personal preferences and self-representations, which is a prerequisite for self-coherent decisions, self-confrontational coping, and mental health [47,50,55,96]. This predisposes individuals with distress-avoidance responses to suffer from pain-related fear/anxiety, hypervigilance, uncontrollable rumination, and helplessness, but also from the consequences of excessive overactivity and frequent failure to perform coping activities that satisfy their needs (alienation) [20,22,41,54].

Prior research additionally supports the assumption of an impaired balance between maintenance of and disengagement from intentions in state-oriented patients with distress-avoidance responses: State orientation was shown to be an organismic risk factor that mutually aggravates deficits in motivation and performance: introjection of social expectations and norms, memory deficits, dissociations between cognitive and emotional preferences, over-activation of intentions, and rigidity against situational changes as well as cognitive and behavioral overactivity [20,22,54,60,68,97,98]. Furthermore, and in line with our results, state orientation was shown to be associated with poor adjustment to chronic pain, helplessness, distress, and depression [20,54,98–102].

One the other hand, we found differences between the pain response profiles in prospective action versus state orientation. Moreover, in comparison to the both other response profiles, patients with distress-avoidance responses suffer from higher levels of pain, life stress, and help-/hopelessness as well as lower health. This may indicate or be a consequence of prolonged exposure to uncontrollable aversive events that may increasingly eliminate perceived action alternatives to successfully cope with pain [50,54,69]. Thus, the ability to up-regulate positive emotions (i.e., self-motivation) and initiate difficult intentions despite pain- and work-related life stress seems most required in patients with distress-endurance responses. Importantly, prospective state orientation may contribute to a lack of initiative in the face of repeated (pain-related) interruptions of ongoing activities and depleted action-facilitating positive emotions [20,47,103]. Hence, apart from fear/anxiety-based escape/avoidance behavior, prospective state orientation may (additionally) reduce pro-/active coping and physical activity, especially in distress-avoidance versus eustress-endurance responders. Likewise, the associated symptoms of procrastination and passivity may be another explanation for the findings that patient groups with diverse (partially confounded) avoidance and/or endurance responses often do not differ from each other in diverse measures of physical activity and/or deconditioning [15,16,43,104].

In comparison to distress-avoidance responders, the findings of more action-oriented eustress-endurance responders (EER; sometimes termed as *doers* or *persistent patients* in other cluster-analytical studies [15,16,18,19]) showed significantly lower levels of life stress, better coping and health. This finding is in line with both FAM and Action Control Theory that propose adaptive patterns of no-fear (self-)confrontational (coping) responses to pain and life stress [25,50]. Consistent with these assumptions, research has found action (vs. state) orientation and eustress-/endurance (vs. distress-/avoidance) responses to be resilience factors, associated with better self/emotion regulation, stress resistance, self-efficacy, health, recovery and/or therapy prognosis [15,16,19,20,22,24,48,51,53,63,65,72,99,105–108]. Thus, in contrast to the assumptions of the AEM, eustress-/endurance responses may not be (longitudinally) dysfunctional as long as they are associated with (action-oriented) flexible goal adjustment and attainment of salient non-pain related goals [17,22,54,109–111]. Together, this can be mostly observed also in the patterns of correlations between action versus state orientation, primary and other secondary measures (see S2 Table).

Likewise, higher levels of endurance responses despite pain may not necessarily indicate overactivity or an imbalance between activity and relaxation [15]. In line with other research, our results rather suggest a more functional approach, which differentiates between more rigid reactive (or “regressive”) versus more flexible proactive (or “progressive”) endurance responses. Whereas reactive pain-related goal pursuit and excessive or pain-contingent persistence were associated with more pain and/or lower health, proactive non-pain goal pursuit and task-contingent persistence were associated with less pain and/or better health [22,23,111,112]. Consistently, task/pain persistence revealed zero correlations with action versus state orientation, physical activity, and mental health (see also S2 Table).

Finally, patients with low-endurance responses (LER) showed lower or equal levels of stress, action versus state orientation, maladaptive coping, and health compared to patients with eustress-endurance responses. Moreover, patients with low-endurance responses reported lower levels of stress, state orientation, maladaptive coping, and health compared to patients with distress-avoidance responses. These findings mostly are in line with our assumptions according to the AEM and/or Action Control Theory, as well as associated research, and suggest a pattern of low-risk-related pain responses, similar to pacing [16,19,20,22,24,26]. Patients with low-endurance responses, however, showed equal or even lower levels of subjective competence and cognitive restructuring compared to distress-avoidance responders. In line with other studies [17,112], this finding contradicts AEM’s assumptions of low-endurance responses/pacing to be a distinct pattern of adaptive pain responses in contrast to both other profiles, at least assessed in patients before an intervention.

Alternatively, in line with our hypotheses according to Action Control Theory, low-endurance responses also may be a result of lower levels of pain- and work-related life stress (i.e., perceived threats and demands). Moreover, the formation and efficacy of low-/avoidance-endurance responses at varying levels of life stress may depend on individual action versus state orientation (e.g., [22,47,50]). Likewise, cognitive-behavioral measures alone may not sufficiently depict a context-adequate balance between activity and relaxation. In line with this notion, McCracken and Samuel [16] as well as Kindermans et al. [112] reported zero or even positive relations between (partially avoidant) activity pacing and depression and/or disability. This suggests a more functional approach to more precisely measuring adaptive pacing in contrast to avoidance by considering action control in the context of varying life stress.

Consistent with Action Control Theory, our results suggest that, at lower levels of pain and life stress, more action-oriented patients with low-endurance responses can better self-regulate their emotions, as well as disengage from unattainable or detrimental goals and failure-related rumination than do patients with distress-avoidance responses. They showed at least similar self-regulatory abilities compared to eustress-endurance responders, who, however, perceive higher levels of pain and life stress. High(er) levels of action orientation at lower levels of (pain-related) life stress result in lower levels of helplessness and depression [20].

However, pain and life stress may vary for each individual and therefore bears the risk that people may temporally be overwhelmed by unexpected higher levels of pain and life stress, associated with a loss of top-down control and lower levels of subjective competence [22,50]. Moreover, stress-dependent regression to lower levels of control means, for example, that less flexible automatized habits, passive or conditioned avoidance responses dominate emotion regulation and coping attempts instead of initiating more difficult intentions to actively cope with pain-related life stress (e.g., to get more physically active and/or distract from pain) [47,50]. A lower flexibility and (long-term) efficiency of patients’ automatized pain responses, and self-/emotion regulation (e.g., pain-contingent activity avoidance vs. flexible persistence and humor/distraction) may additionally contribute to lower subjective competence in low-endurance responders as compared to more active and committed eustress-endurance responders [50,109].

Against this background, the adaptiveness of pacing strategies in the context of varying pain and life stress appears to be a consequence of the efficiency of individual coping habits as well as an adequate balance between intentional maintenance and disengagement facilitated by action (vs. state) orientation [20,22,47,50,67,72]. Future research may address these assumptions on the individual level by considering the role of diathesis-stress interactions in the development of different pain responses with varying degrees of adaptiveness.

### Limitations

Our study was cross-sectional. Therefore, we cannot make any causal conclusions but only generate new hypotheses about the underlying mechanisms of symptom formation. These hypotheses have to be confirmed in future studies. The generalizability of our findings may be limited due to a non-random sample and the exclusion criteria of the primary study that excluded patients with major depression [1]. Results of latent profile analyses further depend on the specific characteristics of analyzed samples and input variables. Thus, results are limited to the population of patients with chronic low back pain and the input measures of the Avoidance-Endurance Questionnaire (AEQ). Further confirmatory research is needed to replicate results, vary and optimize measurements, examine differences between and stability of pain response profiles, and use experimental designs to study underlying mechanisms of symptom chronification and recovery.

### Implications for treatment

In line with other research, our results suggest tailoring of (multiprofessional) interventions based on behavioral and functional analysis of avoidance-endurance responses in order to more effectively improve patients’ quality of life [1,16,22,54,113,114]. More action-oriented patients with eustress-endurance and low-endurance, and most state-oriented patients with distress-avoidance responses demonstrated increasing needs to reduce life stress and improve action control, coping, and health. Distress-avoidance responders may need the most intensive treatment, e.g. mobilizing inpatient rehabilitation programs in combination with after-care to maintain treatment effects. In contrast, eustress-endurance and low-endurance responders may need less intensive treatment to improve coping (abilities) and stabilize patients’ adjustment. Apart from an emerging paradigm shift to the biopsychosocial model and process-based therapy, scientists and clinicians may further develop and evaluate new (interdisciplinary) treatment concepts by considering Action Control Theory [1,47,59,69,106,115]. The measurement of action versus state orientation is recommended (cf.). Diagnostics may additionally reveal frustration of basic needs, discrepancies between explicit goals and implicit motives, cognitive and emotional fixations, and deficits in self-regulatory competencies, which can become subject of individualized therapy and counseling concepts [47,69,74,116,117].

## Conclusions

Our study revealed a new endurance-avoidance typology that was not found in prior studies. We consistently found two distinct, opposite profiles of either distress-avoidance or eustress-endurance responses, and one less distinct and less opposite profile of low- endurance responses in patients with chronic non-specific low back pain. Patients with distress-avoidance responses emerged as the most burdened, dysfunctional patient group concerning measures of stress, action control, maladaptive coping, and health. Compared to both other response profiles, patients with eustress-endurance responses showed one of the highest levels of action versus state orientation, and the highest levels of physical activity. Patients with low-endurance responses reported lower or equal levels of stress, action versus state orientation, maladaptive coping, and health compared to patients with eustress-endurance responses as well as lower or equal low levels of adaptive coping compared to patients with distress-avoidance responses. Results suggest action orientation to be an organismic resilience factor in eustress-endurance and low-endurance responders and state orientation to be an organismic risk factor, especially in distress-avoidance responders. Thereby, the application of Action Control Theory may improve the understanding of various (inconsistent) findings associated with the FAM and AEM as well as the effectiveness of therapy in patients with chronic low back pain.

## Supporting information

S1 Table Bivariate correlations between primary AEQ response measures. Note. AEQ = Avoidance-Endurance Questionnaire; Avoidance-related subscales: C = catastrophizing, HH = help-/hopelessness, AD = anxiety/depression, AP = avoidance of physical activity, AS = avoidance of social activity; Endurance-related subscales: TS = thought suppression, PM = positive mood, HD = humor/distraction, PP = task/pain persistence; *p* = *p*-value, ** = p < .01, * = p < .05; *r* classification of magnitude by Cohen (1988): *r* = .10 small, *r* = .30, medium, *r* = .50, large correlation.
(DOCX)

S2 Table Bivariate correlations between primary and secondary measures. Note. *Primary response measures*: C = catastrophizing, HH = help-/helplessness, AD = anxiety/depression, AP = avoidance of physical activity, AS = avoidance of social activity, TS = thought suppression, PM = positive mood, HD = humor/distraction, PP = task/pain persistence; *Secondary measures*: P = pain, LS = life stress; AOF = failure-related action orientation, AOP = prospective action orientation; SC = subjective competence, CR = cognitive restructuring, PF = pain-related fear (somatic focus), PA = physical activity; D = depression, MH = mental health status, PH = physical health status; *p* = *p*-value, *** = *p* < .001; r classification of magnitude by Cohen (1988) *r* = .10, small, *r* = .30, medium, *r* = .50, large correlation.
(DOCX)

S3 Table Means and standard deviations of pain response profiles in secondary measures. Note: M = unstandardized mean, SD = standard deviation; EER = Eustress-endurance, LER = Low-endurance, DAR = Distress-avoidance responders.
(DOCX)
