# Supplementary material for: Endurance and avoidance response patterns in pain patients: Application of action control theory in pain research
Source: PLoS One. 2021 Mar 25;16(3):e0248875. doi: 10.1371/journal.pone.0248875 (PMC7993813; doi:10.1371/journal.pone.0248875)
Supplement: S1 Table — Note. AEQ = Avoidance-Endurance Questionnaire; Avoidance-related subscales: C = catastrophizing, HH = help-/hopelessness, AD = anxiety/depression, AP = avoidance of physical activity, AS = avoidance of social activity; Endurance-related subscales: TS = thought suppression, PM = positive mood, HD = humor/distraction, PP = task/pain persistence; p = p-value, ** = p < .01, * = p < .05; r classification of magnitude by Cohen (1988): r = .10 small, r = .30, medium, r = .50, large correlation. (DOCX) [file pone.0248875.s001.docx]

Supporting information

**S1 Table. Bivariate correlations between primary AEQ response measures.**

|  | **C** | **HH** | **AD** | **AP** | 1. **AS** | 1. **TS** | 1. **PM** | 1. **HD** | 1. **PP** |
| --- | --- | --- | --- | --- | --- | --- | --- | --- | --- |
| 1. **C** |  |  |  |  |  |  |  |  |  |
| 1. **HH** | .48   1. *** |  |  |  |  |  |  |  |  |
| 1. **AD** | 1. .38 *** | 1. .65 *** |  |  |  |  |  |  |  |
| 1. **AP** | 1. .14 .001 | .26   1. *** | 1. .22 *** |  |  |  |  |  |  |
| 1. **AS** | .29 *** | 1. .44 *** | 1. .49 *** | 1. .59 *** |  |  |  |  |  |
| 1. **TS** | .18 *** | 1. .45 *** | 1. .32 *** | 1. .00 | 1. .14 .001 |  |  |  |  |
| 1. **PM** | -.20 *** | 1. -.38 *** | 1. -.56 *** | 1. -.17 *** | 1. -.42 *** | 1. -.01 |  |  |  |
| 1. **HD** | 1. -.09 .035 | 1. -.22 *** | 1. -.29 *** | 1. -.40 *** | 1. -.45 *** | 1. .19 *** | 1. .52 *** |  |  |
| 1. **PP** | 1. -.02 | .10   1. .024 | 1. -.06 | 1. -.18 *** | 1. -.08 | 1. .59 *** | 1. .22 *** | 1. .47 *** |  |

1. Note. AEQ = Avoidance-Endurance Questionnaire; Avoidance-related subscales: C = catastrophizing, HH = help-/hopelessness, AD = anxiety/depression, AP = avoidance of physical activity, AS = avoidance of social activity; Endurance-related subscales: TS = thought suppression, PM = positive mood, HD = humor/distraction, PP = task/pain persistence; *p* = *p*-value, ** = p < .01, * = p < .05; *r* classification of magnitude by Cohen (1988): *r* = .10 small, *r* = .30, medium, *r* = .50, large correlation.
