# Supplementary material for: Endurance and avoidance response patterns in pain patients: Application of action control theory in pain research
Source: PLoS One. 2021 Mar 25;16(3):e0248875. doi: 10.1371/journal.pone.0248875 (PMC7993813; doi:10.1371/journal.pone.0248875)
Supplement: S2 Table — Note. Primary response measures: C = catastrophizing, HH = help-/helplessness, AD = anxiety/depression, AP = avoidance of physical activity, AS = avoidance of social activity, TS = thought suppression, PM = positive mood, HD = humor/distraction, PP = task/pain persistence; Secondary measures: P = pain, LS = life stress; AOF = failure-related action orientation, AOP = prospective action orientation; SC = subjective competence, CR = cognitive restructuring, PF = pain-related fear (somatic focus), PA = physical activity; D = depression, MH = mental health status, PH = physical health status; p = p-value, *** = p < .001; r classification of magnitude by Cohen (1988) r = .10, small, r = .30, medium, r = .50, large correlation. (DOCX) [file pone.0248875.s002.docx]

1. **S2 Table. Bivariate correlations between primary and secondary measures.**

|  | 1. **P** | 1. **LS** | 1. **AOF** | 1. **AOP** | 1. **SC** | 1. **CR** | 1. **RUM** | 1. **PF** | 1. **PA** | 1. **D** | 1. **MH** | 1. **PH** |
| --- | --- | --- | --- | --- | --- | --- | --- | --- | --- | --- | --- | --- |
| 1. **C** | 1. .09 .035 | 1. .22 2. *** | 1. -.20 *** | 1. -.15 *** | 1. -.12 .007 | 1. -.02 | 1. .40 2. *** | 1. .30 2. *** | 1. -.03 | 1. .20 2. *** | 1. -.25 2. *** | 1. -.05 |
| 1. **HH** | 1. .34 2. *** | 1. .35 2. *** | 1. -.28 2. *** | 1. -.22 2. *** | 1. -.17 2. *** | 1. .02 | 1. .65 2. *** | 1. .49 2. *** | -.12   1. .006 | .39   1. *** | -.39   1. *** | -.28   1. *** |
| 1. **AD** | 1. .30 *** | 1. .46 *** | 1. -.46 *** | 1. -.31 *** | 1. -.27 2. *** | 1. -.04 | 1. .50 2. *** | 1. .35 *** | 1. -.10 2. .021 | 1. .64 2. *** | 1. -.62 2. *** | 1. -.22 2. *** |
| 1. **AP** | 1. .03 | 1. .10 .021 | 1. -.10 .024 | 1. -.14 .001 | 1. -.16 2. *** | 1. -.05 | 1. .27 2. *** | 1. .25 2. *** | 1. -.20 2. *** | 1. .08 2. .064 | 1. -.11 2. .012 | 1. -.18 2. *** |
| 1. **AS** | 1. .13 .003 | 1. .25 *** | 1. -.30 *** | 1. -.18 *** | 1. -.23 2. *** | 1. -.05 | 1. .38 2. *** | 1. .29 2. *** | 1. -.18 2. *** | 1. .31 2. *** | 1. -.38 2. *** | 1. -.20 2. *** |
| 1. **TS** | 1. .20 *** | 1. .22 *** | 1. -.16 *** | 1. -.09 .037 | 1. .14 2. .002 | 1. .28 2. *** | 1. .35 2. *** | 1. .19 2. *** | 1. -.05 | 1. .25 2. *** | 1. -.17 2. *** | 1. -.15 2. .001 |
| 1. **PM** | 1. -.12 .004 | 1. -.23 *** | 1. .38 *** | 1. .27 *** | 1. .40 2. *** | 1. .26 *** | 1. -.30 2. *** | 1. -.28 *** | 1. .12 2. .005 | 1. -.40 2. *** | 1. .45 2. *** | 1. .11 2. .010 |
| 1. **HD** | 1. -.05 | 1. -.09 .041 | 1. .25 *** | 1. .20 2. *** | 1. .43 *** | 1. .30 2. *** | 1. -.23 2. *** | 1. -.21 *** | 1. .22 2. *** | 1. -.20 2. *** | 1. .28 2. *** | 1. .10 2. .028 |
| 1. **PP** | 1. .10 .020 | 1. .13 .003 | 1. -.01 | .05 | 1. .34 *** | 1. .36 2. *** | 1. .04 | 1. -.03 | 1. .01 | 1. .12 2. .006 | 1. .00 | 1. -.12 2. .006 |
| 1. **P** |  | 1. .21 2. *** | 1. -.09 2. .039 | 1. .02 | -.07 | -.00 | 1. .21 2. *** | 1. .11 2. .008 | 1. -.08 | 1. .24 2. *** | 1. -.20 2. *** | 1. -.50 2. *** |
| 1. **LS** |  |  | 1. -.27 2. *** | 1. -.34 2. *** | 1. -.16   *** | -.02 | 1. .20 2. *** | 1. .26 2. *** | 1. -.10 2. .025 | 1. .57 2. *** | 1. -.57 2. *** | 1. -.16 2. *** |
| 1. **AOF** |  |  |  | 1. .54 2. *** | 1. .32   *** | .11  .010 | 1. -.25 2. *** | 1. -.11 2. .013 | 1. .07 | 1. -.42 2. *** | 1. .43 2. *** | 1. .08 |
| 1. **AOP** |  |  |  |  | 1. .31   *** | .10  .021 | 1. -.19 2. *** | 1. -.12 2. .004 | 1. .17 2. *** | 1. -.38 2. *** | 1. .41 2. *** | 1. .01 |
| 1. **SC** |  |  |  |  |  | 1. .61   *** | 1. -.16 2. *** | 1. -.15 2. .001 | 1. .15 2. *** | 1. -.24 2. *** | 1. .33 2. *** | 1. .02 |
| 1. **CR** |  |  |  |  |  |  | 1. .02 | 1. -.03 | 1. .08 | 1. -.06 | 1. .16 2. *** | 1. -.06 |
| 1. **RUM** |  |  |  |  |  |  |  | 1. .42 2. *** | -.12  .006 | .25  *** | -.29  *** | -.19  *** |
| 1. **PF** |  |  |  |  |  |  |  |  | -.15  .001 | .22  *** | -.25  *** | -.18  *** |
| 1. **PA** |  |  |  |  |  |  |  |  |  | -.13  .002 | .14  .001 | .07 |
| 1. **D** |  |  |  |  |  |  |  |  |  |  | -.70  *** | -.19  *** |
| 1. **MH** |  |  |  |  |  |  |  |  |  |  |  | -.04 |
| 1. **PH** |  |  |  |  |  |  |  |  |  |  |  |  |

1. Note. *Primary response measures:* C = catastrophizing, HH = help-/helplessness, AD = anxiety/depression, AP = avoidance of physical activity, AS = avoidance of social activity, TS = thought suppression, PM = positive mood, HD = humor/distraction, PP = task/pain persistence; *Secondary measures:* P = pain, LS = life stress; AOF = failure-related action orientation, AOP = prospective action orientation; SC = subjective competence, CR = cognitive restructuring, PF = pain-related fear (somatic focus), PA = physical activity; D = depression, MH = mental health status, PH = physical health status; *p* = *p*-value, *** = *p* < .001; r classification of magnitude by Cohen (1988) *r* = .10, small, *r* = .30, medium, *r* = .50, large correlation.
