# Supplementary material for: Endurance and avoidance response patterns in pain patients: Application of action control theory in pain research
Source: PLoS One. 2021 Mar 25;16(3):e0248875. doi: 10.1371/journal.pone.0248875 (PMC7993813; doi:10.1371/journal.pone.0248875)
Supplement: S3 Table — Note: M = unstandardized mean, SD = standard deviation; EER = Eustress-endurance, LER = Low-endurance, DAR = Distress-avoidance responders. (DOCX) [file pone.0248875.s003.docx]

**S3 Table. Means and standard deviations of pain response profiles in secondary measures.**

| **Secondary measures** | **EER**  **M (SD)** | **LER**  **M (SD)** | **DAR**  **M (SD)** |
| --- | --- | --- | --- |
| Pain | 5.71 (1.43) | 5.31 (1.78) | 6.18 (1.55) |
| Life stress | 6.89 (5.29) | 5.01 (4.34) | 9.34 (5.26) |
| Failure-related action orientation | 6.43 (3.15) | 6.79 (3.45) | 4.28 (3.16) |
| Prospective action orientation | 7.98 (2.89) | 8.15 (2.81) | 6.38 (3.33) |
| Subjective competence | 18.21 (3.89) | 15.66 (4.58) | 14.76 (4.18) |
| Cognitive restructuring | 15.95 (4.37) | 12.65 (5.05) | 13.96 (4.17) |
| Rumination | 1.82 (0.76) | 1.57 (0.78) | 2.62 (0.72) |
| Pain-related fear (somatic focus) | 4.11 (3.05) | 3.84 (2.97) | 6.62 (3.39) |
| Physical activity (h) | 9.46 (5.59) | 8.02 (6.11) | 6.87 (5.36) |
| Depression | 5.48 (3.36) | 4.44 (3.21) | 8.79 (4.68) |
| Mental health | 50.52 (9.59) | 51.44 (9.87) | 41.14 (10.96) |
| Physical health | 38.77 (8.66) | 39.02 (9.20) | 35.89 (7.94) |

1. Note: M = unstandardized mean, SD = standard deviation; EER = Eustress-endurance, LER = Low-endurance, DAR = Distress-avoidance responders.
